# A Mechanistic, Architecture-Dependent Study Combining Experiments and Molecular Dynamics to Explain AMP Release from GO–PEI Coatings

**DOI:** 10.3390/bioengineering13030341

**Published:** 2026-03-15

**Authors:** Adriana de América, María José Fritte, Paola Alarcón, Karel Mena-Ulecia, Gonzalo Recio-Sánchez, Klaus Rischka, Marcos Rocha Diniz Silva, Matheus Santos Dias, Camila Marchetti Maroneze, Cecilia de Carvalho Castro Silva, Jacobo Hernandez-Montelongo

**Affiliations:** 1 Departamento de Ciencias Físicas y Matemáticas, Universidad Católica de Temuco, Temuco 4813302, Chile; adeamerica2017@alu.uct.cl (A.d.A.); mfritte2018@alu.uct.cl (M.J.F.); paola.alarcon2015@alu.uct.cl (P.A.); 2 Núcleo de Investigación en Bioproductos y Materiales Avanzados (BioMA), Universidad Católica de Temuco, Temuco 4813302, Chile; kmena@uct.cl; 3 Facultad de Ingeniería, Arquitectura y Diseño, Universidad San Sebastián, Concepción 4080871, Chile; g.recio.sanchez@gmail.com; 4 Department of Adhesive Bonding Technology and Surfaces, Fraunhofer Institute for Manufacturing Technology and Advanced Materials (IFAM), 28359 Bremen, Germany; klaus.rischka@ifam.fraunhofer.de; 5 Mackenzie School of Engineering, Mackenzie Presbyterian University, São Paulo 01302-907, Brazil; marcosrochadiniz@hotmail.com (M.R.D.S.); theeus.santos@gmail.com (M.S.D.); camila.maroneze@mackenzie.br (C.M.M.); cecilia.silva@mackenzie.br (C.d.C.C.S.); 6 MackGraphe-Mackenzie Institute for Research in Graphene and Nanotechnologies, Mackenzie Presbyterian University, São Paulo 01302-907, Brazil

**Keywords:** urinary catheters, graphene oxide, antimicrobial peptides, molecular dynamics, coatings

## Abstract

This study investigates two graphene oxide (GO)-based coating architectures on urinary catheter substrates—layered (PEI+GO) and embedded (PEI/GO)—loaded with antimicrobial peptides (E14LKK and fLFB), with the aim of elucidating how coating structure governs peptide retention and release. Physicochemical and morphological characterization confirmed distinct coating architectures and thicknesses. Molecular dynamics simulations were employed to probe GO–peptide and PEI–peptide interactions, revealing weaker binding of fLFB to GO relative to PEI, consistent with enhanced peptide mobility. Antibacterial performance against *Escherichia coli* and *Enterococcus faecalis* was evaluated using agar diffusion assays as a comparative indicator of peptide release from surface-bound coatings. The layered PEI+GO–fLFB system exhibited the highest antibacterial activity, in agreement with simulation-predicted interaction energetics and structural fluctuations. Rather than targeting immediate clinical translation, this work provides mechanistic insight into how GO–polymer architecture modulates antimicrobial peptide availability, offering a molecular dynamics simulation-guided framework for the rational design of peptide-releasing antimicrobial coatings.

## 1. Introduction

Urinary catheters (UCs) are commonly used for urine drainage in patients with urinary obstructions, post-surgical recovery, or kidney failure. However, prolonged catheterization significantly increases the risk of catheter-associated urinary tract infections (CAUTIs), which account for 20–30% of healthcare-associated infections (HAIs) and are linked to elevated mortality rates and healthcare costs [1,2,3]. A key challenge in CAUTI management is the formation of bacterial biofilms on catheter surfaces, which diminishes antibiotic efficacy and complicates treatment [2].

To overcome this issue, antibacterial nanomaterials have emerged as promising candidates due to their high surface area and tunable physicochemical properties [4]. Among them, graphene oxide (GO), a two-dimensional carbon-based nanomaterial, has demonstrated notable antibacterial activity [5,6,7,8]. Its large surface area and hydrophilicity make it especially suitable for use in coatings and as a carrier for drugs and biomolecules [9,10]. GO’s integration with polyethyleneimine (PEI), a cationic polymer, facilitates surface deposition through electrostatic interactions and enhances coating stability [11].

The incorporation of antimicrobial peptides (AMPs) offers an additional antibacterial mechanism. AMPs are short peptides with broad-spectrum antimicrobial activity that act by disrupting bacterial membranes and intracellular processes, while exhibiting low cytotoxicity and reduced potential for resistance development [12]. In this study, two AMPs were selected: E14LKK, a magainin-derived α-helical peptide [13], and fLFB, a fragment of lactoferricin B with broad antimicrobial efficacy [14].

Despite the growing interest in GO-based antimicrobial coatings for urinary catheters, a fundamental understanding of how coating architecture influences antimicrobial peptide retention and release remains limited. Many previous studies emphasize antibacterial performance alone, often without correlating experimental outcomes with the molecular-scale interactions that govern peptide availability at the material interface. As a result, design principles linking coating structure to functional performance remain poorly defined.

In this context, the present study does not aim to deliver a clinically validated catheter coating. Instead, it focuses on elucidating the structure–function relationships between GO–polymer architecture and antimicrobial peptide release through a combined experimental and molecular dynamics approach. Two GO-based coating configurations were investigated: a layered structure consisting of PEI followed by GO (PEI+GO) and an embedded architecture combining PEI and GO (PEI/GO). Both systems were loaded with either E14LKK or fLFB. The coatings were characterized in terms of their physicochemical and morphological properties, while GO–AMP and PEI–AMP interactions were examined using molecular dynamics simulations. Antibacterial performance was evaluated against *Escherichia coli* (Gram-negative) and *Enterococcus faecalis* (Gram-positive), two clinically relevant pathogens associated with CAUTIs [15,16].

## 2. Materials and Methods

### 2.1. Materials

A 20 Fr silicone Foley urinary catheter (UC) was purchased from Chen Kang (Yichun, China). Polyethyleneimine (PEI, 50 wt.% in water, MW ≈ 7.5 × 10^5^ g/mol) was obtained from Sigma-Aldrich (St. Louis, MO, USA). Ethanol, NaOH, and HCl were from Merck (Santiago, Chile), while H_2_SO_4_, KMnO_4_, and H_2_O_2_ were from Synth (Sao Paulo, Brazil). NaNO_3_ was acquired from Merck (Darmstadt, Germany). All reagents were used as received, and solutions were prepared with Milli-Q water (18.2 MΩ·cm, pH 7.6 unless stated otherwise).

### 2.2. Graphene Oxide Synthesis

Graphene oxide (GO) was synthesized via a modified Hummers’ method [17], with a total oxidation time of three days and purification as described by Rocha [18]. Graphite flakes (0.5 g) were stirred with 16.9 mL H_2_SO_4_ in an ice bath for 30 min, followed by gradual addition of 2.25 g KMnO_4_ over 1 h. The mixture was stirred for 24 h and left at room temperature for 72 h. After oxidation, 50 mL of 0.06 mol/L H_2_SO_4_ was added over 1 h, followed by 1.5 mL of 30% H_2_O_2_. The GO was washed with 10% HCl and dialyzed (12 kDa, Sigma-Aldrich; St. Louis, MO, USA) in distilled water until pH 5.5, then concentrated to 5.80 mg/mL using a rotary evaporator (Q344M, QUIMIS; Diadema, Brazil).

### 2.3. Solid-Phase Peptide Synthesis

The antimicrobial peptides E14LKK (LKLLKKLLKLLKKL) and fLFB (RRWQWRMKKLG) were synthesized using a microwave-assisted synthesizer (Liberty Blue, CEM; Kamp-Lintfort, Germany) with Fmoc-protected amino acids (IRIS Biotech; Marktredwitz, Germany) on Fmoc-Rink-Amide resin (0.30 mmol/g, INTAVIS; Tübingen, Germany). Assembly was conducted on a 0.1 mmol scale in DMF using DIC/Oxyma (0.5/1.0 M). Fmoc deprotection was done with 20% piperidine in DMF. Cleavage used 95% TFA, 2.5% water, and 2.5% triisopropylsilane, followed by precipitation in cold methyl tert-butyl ether, solubilization in UHQ water, and freeze-drying. Molecular weights were 1692.31 g/mol (E14LKK) and 1544.87 g/mol (fLFB), with ≥95% purity confirmed by MALDI-ToF MS (Autoflex Speed, Bruker; Billerica, MA, USA). 3D structures were modeled using UCSF Chimera v1.14.

### 2.4. Coating of Samples

**Figure 1:**
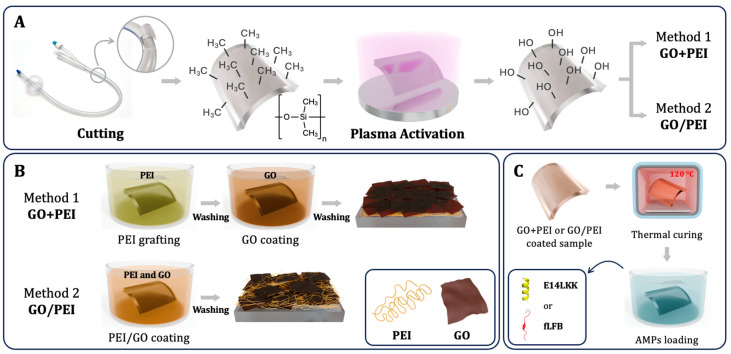
Schematic of the coating process: (**A**) Plasma pretreatment, (**B**) Coating via 1. PEI+GO or 2. PEI/GO, and (**C**) Thermal curing and AMPs (E14LKK or fLFB) loading. The scheme is illustrative; chemical changes are idealized.

Catheter samples (0.5 cm, ∅ = 6.7 mm) were cut from a 20 Fr Foley silicone UC, rinsed with ethanol, dried, and activated by O_2_ plasma treatment (for 15 min, under a pressure lower than 0.2 mmHg and power of 18 W, Harrick Plasma; Ithaca, NY, USA) (Figure 1A). Two coating methods were used: PEI+GO and PEI/GO (Figure 1B). In PEI+GO, pretreated samples were immersed in PEI (1 mg/mL, pH 4) for 15 min, rinsed, and then coated with GO (0.1 mg/mL, pH 10). In PEI/GO, samples were directly immersed in a mixed PEI/GO solution (0.1 mg/mL, pH 7). Both were thermally cured at 120 °C for 1 h (Figure 1C). After cooling, AMPs (E14LKK or fLFB) were loaded by physical adsorption (0.5 mg/mL, 1 h).

### 2.5. Characterization Techniques

Zeta potentials of GO, PEI, and PEI/GO solutions were measured in triplicate using a Zetasizer Advance (Malvern; Worcestershire, UK). Wettability of coated UC samples was assessed via static sessile drop (10 μL, n = 5) using a Droplet Lab system (Brampton, ON, Canada). Surface roughness (RRMS) was measured with a PCE-RT 2300 tester (PCE Instruments; Southampton, UK) per ISO 4287 [19]. FTIR spectra were collected in transmittance mode (4600, Jasco; Tokyo, Japan; 4000–600 cm^−1^, 1 cm^−1^ resolution, NS = 4) and processed by smoothing and normalization. Raman spectra were obtained at room temperature (785 nm laser, 300–2000 cm^−1^, Dual Hound, Unchained Labs; Pleasanton, CA, USA). Morphology was analyzed by optical microscopy (Eclipse E200; Nikon, Tokyo, Japan), VP-SEM (SU-3500, Hitachi; Tokyo, Japan), and FEG-SEM (JSM-7800, Jeol; Tokyo, Japan) for GO sheet dimensions.

### 2.6. Molecular Dynamics Simulations

To study GO–peptide interactions, a computational protocol was applied. A 20 × 20 Å^2^ GO sheet with OH, COOH, and epoxide groups was built in VMD [20] and Chemcraft [21], then optimized using B3LYP/DEF2-TZVPP in Orca [22,23,24] and validated via frequency analysis. Peptides E14LKK and fLFB (from PDB: 1LFC) [13,25] were prepared in UCSF Chimera [26], hydrogenated at pH 7.4. The structure of PEI was downloaded from the PubChem database, its code is CID-170907045, and was hydrogenated to pH 4.0 using the Avogadro 2.0 program [27]. The sheet of this material was constructed with 4 PEI subunits using the same program. Each GO-AMP and PEI–AMP complex was solvated in a TIP3P water box [28,29], and GO and PEI parameters were assigned via SwissParam [30], with CGenFF [31] and CHARMM36 [32] for GO, PEI, and AMPs. Systems underwent 20,000-step minimization (steepest descent) [33], followed by 2 ns equilibration and 50 ns production (300 K, weak coupling, 12 Å cutoff, PME [34], Verlet integrator, 1 fs step) using NAMD 2.13 [35,36,37].

### 2.7. Bacterial Assays

The antibacterial activity of GO-coated samples loaded with antimicrobial peptides (AMPs) was tested against *E. coli* and *E. faecalis* using the agar diffusion method on Mueller–Hinton agar. Bacterial suspensions (0.5 McFarland) were inoculated, and samples were placed vertically on the agar surface, dried for 1 h, and incubated at 35 °C for 24 h. Inhibition zones were measured and normalized using [38]:

(1) $${nw}_{halo}=\;\frac{\frac{d_{iz}-d}{2}}{d}$$

where *nw_halo_* is the normalized width of the antimicrobial “halo”, the diameter of the inhibition zone is *d_iz_*, and the diameter of the catheter is *d.* Data were analyzed using analysis of variance (ANOVA) followed by a Tukey post hoc test in OriginLab v.2022b. Significant differences were presented as * is *p*-value ≤ 0.05, ** is *p*-value ≤ 0.01, and *** is *p*-value ≤ 0.001. All assays were performed in triplicate.

## 3. Results and Discussion

Samples were obtained from commercial 20 Fr silicone Foley UCs, composed mainly of hydrophobic polydimethylsiloxane (PDMS) [39]. To enhance coating adhesion, surfaces were pretreated with oxygen plasma to introduce silanol groups [40] (Figure 1A). Two coating strategies were then applied: PEI+GO and PEI/GO (Figure 1B). In the PEI+GO cascade method, a PEI layer (pH 4, ζ = +43.2 ± 7.2 mV) was deposited first, followed by GO (pH 10, ζ = −31.5 ± 1.4 mV) [41,42,43]. The electrostatic interaction between layers enabled stable GO attachment. In the PEI/GO method, PEI and GO were premixed at pH 7 (ζ = +29.8 ± 0.9 mV), enabling interaction with the negatively activated UC surface. Both coatings were thermally cured at 120 °C for 1 h (Figure 1C) to improve adhesion, stability, and facilitate AMPs loading. All assays used cylindrical catheter sections (0.5 cm); the rectangular shape in Figure 1 is a schematic reference only.

**Figure 2:**
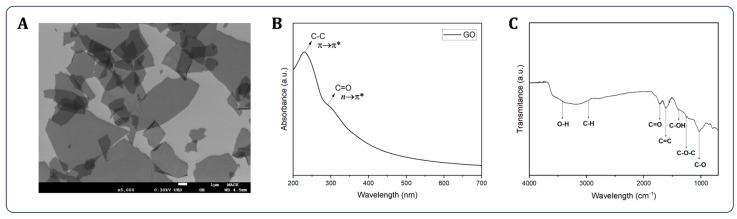
Characterization of GO by: (**A**) scanning electron microscopy, (**B**) UV-Vis spectroscopy, and (**C**) FTIR analysis.

Figure 2 presents the physicochemical characterization of GO. The monolayer flakes exhibited lateral dimensions ranging from 1 to 10 µm (Figure 2A). The UV–Vis spectrum (Figure 2B) showed a characteristic absorption peak at 230 nm corresponding to the π→π* of C–C bonds and a shoulder at 300 nm attributed to *n*→π* of C=O groups [44]. The FTIR spectrum (Figure 2C) confirmed the presence of various oxygen-containing functional groups, including O–H (3420 cm^−1^), C–H (2966 cm^−1^), C=O (1722 cm^−1^), C=C (1615 cm^−1^), C–OH (1388 cm^−1^), C–O–C (1259 cm^−1^), and C–O (1034 cm^−1^) [45,46]. 

**Figure 3:**
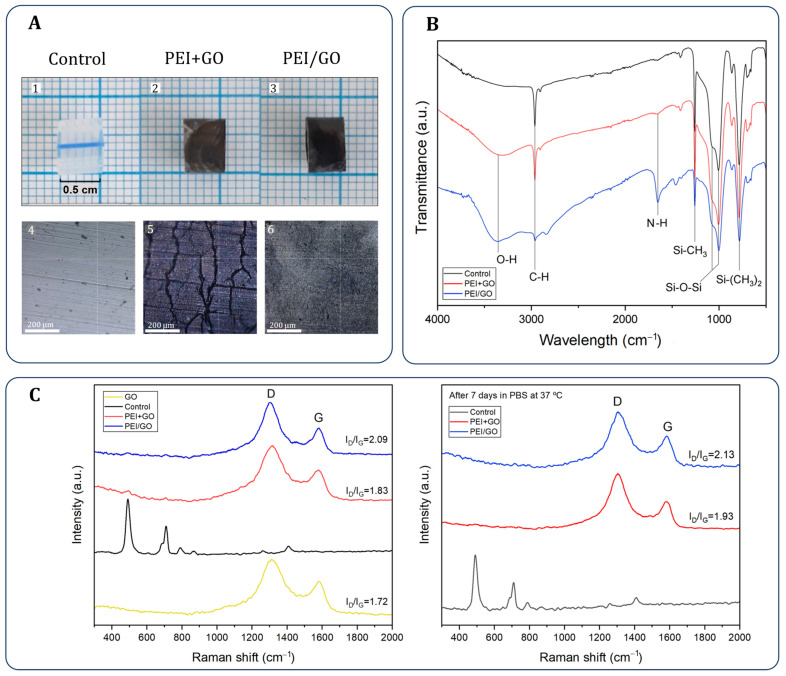
Physicochemical characterization of GO-coated samples: (**A**) images and microscopy, (**B**) contact angle and roughness, (**C**) FTIR spectra, and Raman spectra before and after 7 days in PBS at 37 °C.

Figure 3A displays uniform GO coatings achieved by both deposition methods. Microscopy revealed that the PEI+GO surface exhibited a porous and cracked morphology, while the PEI/GO coating appeared smoother—likely due to improved polymer integration. FTIR analysis of the coated urinary catheters (UCs) (Figure 3B) confirmed characteristic PDMS signals, including Si–(CH_3_)_2_ at 784 cm^−1^, Si–O–Si at 1007 and 1068 cm^−1^, Si–CH_3_ at 1257 cm^−1^, and CH_3_ at 2962 cm^−1^ [47]. Additional absorption bands corresponding to GO and PEI were also observed, such as N–H bending at 1675 cm^−1^ [48] and a broad O–H stretching band between 3100 and 3500 cm^−1^ [47,49], with greater intensity in the PEI/GO sample—suggesting a higher or more exposed PEI content. Figure 3C presents Raman spectra: control UCs (pristine PDMS) exhibited characteristic peaks at 492 and 710 cm^−1^ (Si–O–Si and C–Si–C, respectively) [50], while GO-coated samples displayed the typical D and G bands at ~1350 and ~1580 cm^−1^. The intensity ratio (I_D_/I_G_) increased from 1.72 in pure GO to 1.83 in PEI+GO and 2.09 in PEI/GO, indicating increased structural disorder attributed to the reducing and modifying effects of PEI [51]. Figure 3C also shows the Raman spectra of the samples after exposure to PBS solution (pH 7.4, 37 °C) for 7 days, intended to assess the stability of the coatings under conditions that simulate physiological fluids. As observed, the spectra remain largely unchanged compared to the pre-immersion samples, with only a slight increase in the I_D_/I_G_ ratio. This subtle variation is probably due to interactions between PBS ions and the oxygen-containing functional groups present in GO. Finally, both samples exhibited increased surface roughness and hydrophilicity as a result of the coating processes (Table 1).

**Figure 4:**
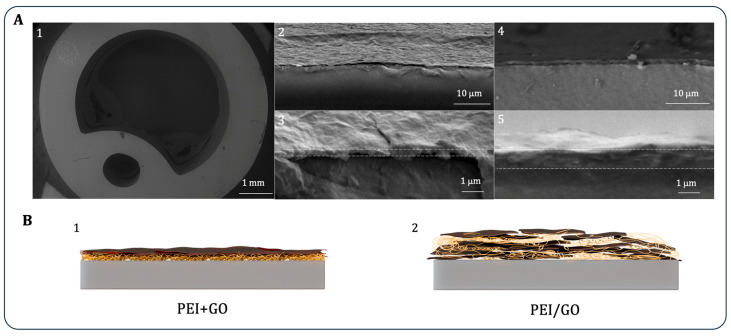
Cross-sectional SEM images of 20Fr control UC sample (**A**) (**1**), PEI+GO (**2**,**3**), and PEI/GO coatings (**4**,**5**). Schematics of PEI+GO (**B**) (**1**) and PEI/GO (**2**) coatings.

Figure 4A presents cross-sectional SEM images of both control and GO-coated samples. The uncoated UC (Figure 4A(1)) exhibits an outer diameter of 6.7 mm and an inner diameter of 4.7 mm, in accordance with the manufacturer’s specifications. The PEI+GO coating (Figure 4A(2,3) reveals a folded, corrugated morphology characteristic of GO layers [52], with an average thickness of 320 ± 33 nm. In contrast, the PEI/GO coating (Figure 4A(4,5)) appears smoother and more homogeneous, with a significantly greater thickness of 890 ± 115 nm—nearly three times thicker—resulting from the incorporation of GO within the PEI matrix. The structural differences are further illustrated in the schematics of Figure 4B, which depict the layered configuration of PEI+GO (Figure 4B(1)) and the embedded structure of PEI/GO (Figure 4B(2)).

**Figure 5:**
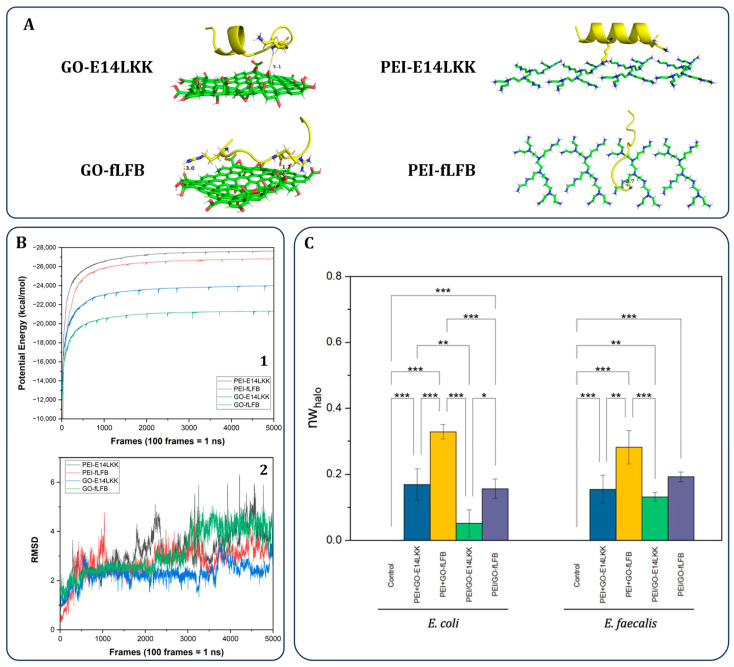
Molecular dynamics and antibacterial performance. (**A**) GO–AMPs and PEI–AMP complexes. (**B**) (**1**) Potential energy profiles over 50 ns. (**2**) RMSD behavior of complexes. (**C**) Antibacterial activity against *E. coli* and *E. faecalis*. Data are mean ± SD (*n* = 3), analyzed by ANOVA with Tukey post hoc test. Significance: * is *p*-value ≤ 0.05, ** is *p*-value ≤ 0.01 and *** is *p*-value ≤ 0.001.

Molecular dynamics (MD) simulations (Figure 5A,B) further analyzed the stability of these complexes during 50 ns of simulation time. Figure 5A shows representative 3D images of the studied complexes: GO-E14LKK, GO-fLFB, PEI-E14LKK, and PEI-fLFB. One of the important parameters for analyzing the stability of the complexes is the potential energy of the systems. As can be seen in Figure 5B(1), the complexes stabilized from the 8 ns simulation time onwards, with the most negative energies corresponding to the complexes formed by PEI and the peptides E14LKK and fLFB. This indicates that these complexes remain more stable over time, and therefore, the peptides will be released less into the PEI matrix than GO. In that sense, the fact that the GO–fLFB complex maintained the highest potential energy is consistent with a less favorable and stable interaction compared to the other complexes.

Another important parameter for analyzing the stability of the studied complexes over time is the root-mean-square deviation (RMSD). As can be seen in Figure 5B(2), this parameter fluctuated significantly in all four studied complexes, with mean RMSD values greater than 3 Å, indicating that GO and PEI matrices could be suitable for the release of AMPs. However, the greatest fluctuations in this parameter were observed with the GO-fLFB complex, indicating that this system behaved unstably over time. Therefore, we can conclude that fLFB will be released more readily in this matrix. It is important to note that in this work, our molecular dynamics simulations focused specifically on the GO–AMPs and PEI–AMP complexes, rather than the full coating architectures (PEI+GO–AMPs or PEI/GO–AMPs). This targeted approach isolates the key interactions between the peptides and the individual coating components (GO and PEI), which primarily govern antimicrobial efficacy by influencing peptide release and bioavailability.

Antibacterial activity (Figure 5C) was evaluated using agar diffusion assays against *E. coli* (Gram-negative) and *E. faecalis* (Gram-positive). Although antimicrobial peptides (AMPs) generally exhibit limited diffusivity in agar-based systems [47], the PEI+GO–fLFB coating produced the largest inhibition zones. This enhanced activity is attributed to two main factors: (i) the weaker interaction between GO and fLFB, which facilitates peptide release, in contrast to the stronger binding of the other complexes that restricts their diffusion; and (ii) the coating architecture—PEI+GO outperformed PEI/GO, likely because the embedded GO in PEI/GO hampers AMPs loading and release due to screening of GO’s functional groups. Among all tested samples, PEI/GO–E14LKK exhibited the weakest antibacterial activity, highlighting the critical role of both the coating configuration and the strength of GO–AMP interactions in achieving effective antibacterial performance on UCs.

## 4. Conclusions

This study developed two GO-based coating strategies for UCs using PEI: a layered PEI+GO architecture and an embedded PEI/GO configuration, both incorporating antimicrobial peptides (AMPs) E14LKK and fLFB to prevent catheter-associated urinary tract infections (CAUTIs). The PEI+GO approach resulted in a porous 320 nm coating, while the PEI/GO method produced a smoother, thicker layer (~890 nm) due to the integration of GO within the PEI matrix. Antibacterial assays against *E. coli* and *E. faecalis* revealed that PEI+GO–fLFB exhibited the highest efficacy, likely due to weaker GO–fLFB interactions that facilitated peptide release—an effect corroborated by molecular dynamics simulations. In contrast, the PEI/GO coatings demonstrated reduced antibacterial activity, attributed to limited AMPs availability. These findings underscore the importance of both coating architecture and AMPs–GO interaction strength in dictating antimicrobial performance. Future studies should evaluate the coated urinary catheters using complementary antibiofilm assays and expand molecular dynamics simulations to encompass the complete coating architectures (PEI+GO and PEI/GO) with both antimicrobial peptides.

## Figures and Tables

**Table 1:** Water contact angle and surface roughness of samples.

Sample	Water Contact Angle (°)	R_RMS_ (μm)
Control	98.3 ± 1.0	0.16 ± 0.03
PEI+GO	76.4 ± 1.0	0.25 ± 0.03
PEI/GO	75.5 ± 1.0	0.28 ± 0.03

## Data Availability

The dataset is available on reasonable request from the authors.
